# The Viral Susceptibility of the *Haloferax* Species

**DOI:** 10.3390/v14061344

**Published:** 2022-06-20

**Authors:** Zaloa Aguirre Sourrouille, Sabine Schwarzer, Sebastian Lequime, Hanna M. Oksanen, Tessa E. F. Quax

**Affiliations:** 1 Biology of Archaea and Viruses, Groningen Biomolecular Sciences and Biotechnology Institute, University of Groningen, 9747 AG Groningen, The Netherlands; z.aguirre.sourrouille@rug.nl (Z.A.S.); s.schwarzer@rug.nl (S.S.); 2 Archaeal Virus-Host Interactions, Faculty of Biology, University of Freiburg, 97104 Freiburg im Breisgau, Germany; 3 Cluster of Microbial Ecology, Groningen Institute for Evolutionary Life Sciences, University of Groningen, 9747 AG Groningen, The Netherlands; s.j.j.lequime@rug.nl; 4 Molecular and Integrative Biosciences Research Programme, Faculty of Biological and Environmental Sciences, University of Helsinki, 00014 Helsinki, Finland

**Keywords:** haloarchaea, archaeal virus, *Haloferax*, *Haloferax gibbonsii* LR2-5, host range

## Abstract

Viruses can infect members of all three domains of life. However, little is known about viruses infecting archaea and the mechanisms that determine their host interactions are poorly understood. Investigations of molecular mechanisms of viral infection rely on genetically accessible virus–host model systems. Euryarchaea belonging to the genus *Haloferax* are interesting models, as a reliable genetic system and versatile microscopy methods are available. However, only one virus infecting the *Haloferax* species is currently available. In this study, we tested ~100 haloarchaeal virus isolates for their infectivity on 14 *Haloferax* strains. From this, we identified 10 virus isolates in total capable of infecting *Haloferax* strains, which represented myovirus or siphovirus morphotypes. Surprisingly, the only susceptible strain of all 14 tested was *Haloferax gibbonsii* LR2-5, which serves as an auspicious host for all of these 10 viruses. By applying comparative genomics, we shed light on factors determining the host range of haloarchaeal viruses on *Haloferax*. We anticipate our study to be a starting point in the study of haloarchaeal virus–host interactions.

## 1. Introduction

Microbial viruses are widespread and able to infect members of all three domains of life, including archaea. Archaea are ubiquitous microorganisms that can be found in extreme environments, such as salt lakes as well as in mesophilic surroundings such as the oceans and the human body [1,2]. The study of archaeal viruses is essential to understand the origin and the evolution of viruses in general [3]. Archaeal viruses display a high genomic and structural variability, but they also share some common traits with viruses infecting other domains of life [4,5,6,7]. Viruses are divided into different families, currently mainly based on the sequence similarities and the highest taxonomic ranks; realms largely follow groupings based on the characteristics of major virus capsid proteins or genome replication components [5,8,9]. Whereas crenarchaeal viruses come in many different shapes, the majority of viruses infecting euryarchaea display a head–tail morphology and are currently members of 14 families in the class *Caudoviricetes* [6,9,10,11,12]. The rest of the currently known euryarchaeal viruses are either internal membrane-containing tailless icosahedral (family *Sphaerolipoviridae*) [13], pleomorphic (family *Pleolipoviridae*) [14], or spindle-shaped (family *Halspiviridae*) [15]. Archaeal tailed viruses are the most common isolates infecting halophilic archaea. They are morphologically indistinguishable from tailed double-stranded (ds) DNA bacteriophages that have the myovirus (long and contractile helical tail), podovirus (short tails), or siphovirus (long and non-contractile tails) morphology. At the sequence level, however, archaeal tailed viruses are very diverse, several of them are singletons, and they hardly resemble their bacterial relatives [10,16]. All isolated archaeal viruses have a DNA genome so far, and the majority of their genes encode proteins of unknown function showing limited or nonexistent similarity to tailed bacteriophage proteins, as a result many aspects of archaeal virus–host relationships and virus life cycles remain unknown [6]. However, very recently, comparative genomics and a host range analysis of the tailed archaeal viruses showed the role of the tail fiber adhesin in host recognition [10]. The adhesins from archaeal viruses resemble the adhesins located at the distal tip of the tail fibers of various members of the T-even phage group [17]. The structural core of the adhesins is formed by highly conserved glycine-rich motifs that separate the hypervariable segments [17]. While these conserved glycine-rich domains are used for binding, mutations or shuffling of the hypervariable regions change adhesin receptor specificity and thus primarily determine the host range [10,18]. Previous screenings of haloarchaeal viruses and their hosts highlighted the high abundance of myovirus isolates and their extremely broad host ranges. The *Hafunaviridae* is the largest family of archaeal tailed viruses and its myoviruses have a broad host range [10,19]. Other haloarchaeal viruses are more specific to a certain host [10,18].

The study of the virus–host relationships and infection mechanisms of archaeal viruses would greatly benefit from the availability of genetically accessible virus–host models, for which molecular biology tools are available. For crenarchaeal virus hosts, several members of the genus *Sulfolobales* are currently the most common archaeal models with genetic systems available [20,21,22,23,24]. Moreover, there are already some genetic systems for crenarchaeal viruses available such as SSVs, STIV, and STSV1 [25,26,27,28]. The study of euryarchaeal viruses presently relies mainly on haloarchaeal *Halorubrum* and *Haloarcula* strains that are infected by a substantial number of known viruses [10,19,29]. These hosts have been used successfully to study archaeal viruses and their structures, entry, and egress mechanisms [30,31,32,33,34]. However, the genetic and the molecular toolset for these hosts is limited. In recent years, *Haloferax* has become increasingly popular in the archaeal scientific community, and it is the euryarchaeal model for which the most advanced tools for genetic engineering, imaging, and molecular biology are available [35]. The *Haloferax* tools entail a versatile genetic system for overexpression and genomic knock-out, the availability of several plasmids and different markers, and a CRISPR-based repression system to downregulate gene expression [23,36,37,38,39]. In addition, it is the only archaeon for which several fluorescent fusion proteins are available [40]. It is an excellent organism for light microscopy, and it is also used in microfluidics [40]. The development of these technical advances, and the growing scientific community embracing *Haloferax* as a central euryarchaeal model, has led to a substantial increase in the understanding of its cell biology [41,42,43,44,45,46,47,48,49,50,51,52,53,54,55,56]. This detailed knowledge of euryarchaeal cell biology, obtained using *Haloferax* as a model, is of great added value in studies of viral infection mechanisms.

In contrast to some other haloarchaea, there are far less viruses known that infect *Haloferax* strains. Almost 30 years ago, the HF1 virus was reported to infect *Haloferax lucentense* and *Hfx. volcanii*, and a defective provirus of *Haloferax mediterranei* has been identified, both of which are no longer available (M. Dyall-Smith, personal communication) [57,58]. At the moment, Haloferax tailed virus 1 (HFTV1) is only one virus isolated from a *Haloferax* host, and it was recently isolated together with its host *Haloferax gibbonsii* LR2-5 from the hypersaline Lake Retba in Senegal [59,60].

Due to the attractiveness of *Haloferax* for molecular studies, we aim to identify viruses that infect *Haloferax* strains. In this study, we used the largest available collection of isolated and characterized haloarchaeal virus isolates, and we tested the infectivity of 95 viruses on 14 *Haloferax* strains. The virus collection contains viruses from all current haloarchaeal virus families, and these viruses represent the majority of haloarchaeal virus isolates isolated to date. This endeavor resulted in an extended virus–host matrix for *Haloferax* from which we could identify a promising model that is the host to a substantial number of viruses, *Haloferax gibbonsii* LR2-5. In addition, we used comparative genomics to identify viral factors that allow for the infection of this *Haloferax* host. With this work, we pave the way to using *Haloferax* as a model in virus–host interactions.

## 2. Materials and Methods

### 2.1. Archaeal Viruses and Strains and Growth Conditions

All *Haloferax* strains (Supplementary Table S1), virus host strains (Supplementary Table S2), and viruses (Supplementary Table S2) were grown aerobically at 37 °C in a modified growth medium (MGM) [58,61]. The artificial 30% salt water (SW) (240 g NaCl, 30 g MgCl_2_ ·6H_2_O, 35 g MgSO_4_ · 7H_2_O, 7 g KCl, 5 mL of 1 M CaCl_2_ · 2H_2_O, and 80 mL of 1 M Tris-HCl, pH 7.2 per L) was diluted to obtain 18, 20, or 23% SW in the top-agar media, plates, and liquid media, respectively. MGM also contained 5 g of peptone (Oxoid), 1 g of Bacto yeast extract (Becton, Dickinson and Company, Sparks, MD, USA), and Bacto agar (14 g for plates; 4 g per top-layer; Becton, Dickinson and Company) per liter. Viruses were grown on their own host strain listed in Supplementary Table S2 by using a double-layer plaque assay. For plaque assays, the strains were grown over 2–3 nights in liquid media to obtain a dense culture (OD~1) of which 200–300 µL were used per plate for inoculation to obtain an even layer of dense growth of the strain on a soft agar layer. Viruses diluted in MGM broth were added 100 µL per plate. The virus and the strain were combined with melted soft agar (3–4 mL per plate; 50 °C), mixed, and plated. The plates were grown for 2–4 days in a box (in some cases with an additional cup of water) to prevent them from drying out. The plaques observed on plates were used to enumerate the viruses in the samples by taking into account the virus dilution, and plates with confluent or semi-confluent growth of virus were used to produce virus lysates. Virus lysates were prepared by mixing the collected soft agar layer from confluent or semi-confluent plates with 2–3 mL of MGM broth per plate. After 1.5 h of shaking at 37 °C, lysates were cleared from cell debris and agar by centrifugation (Sorvall F14 rotor, 10,000 rpm, 20 min, 4 °C). Lysates were stored at +4 °C for up 2 months before use.

### 2.2. Sensitivity of Haloferax Strains to Euryarchaeal Viruses

The sensitivity of *Haloferax* strains (Supplementary Table S1) to viruses (Supplementary Table S2) was determined by placing 10 µL drops of undiluted and 1:100 diluted virus lysates applied to *Haloferax* strain plated with top-agar on a plate using a double-layer plaque assay. The host strains of the viruses (Supplementary Table S2) were used as positive controls. For virus drops, MGM was used as a negative control. The drops were repeated in duplicate. All cases where cell growth was inhibited on a plate in the presence of virus lysate were further tested by a double-layer plaque assay to confirm viral infection and to obtain the numerical values of efficiency of infection. All titer data was collected at 37 °C.

### 2.3. Viral Comparative Genomics

The genomic sequences of the viruses were retrieved from NCBI [62]. Phylogenetic trees were constructed using a custom-written R script with the package ggtree [63], using the ANI values calculated with VIRIDIC [64]. Whole genome sequence alignments were visualized with Easyfig [65]. To find putative virus–host determinants, protein sequences found at variable regions of the viral genomes were downloaded from NCBI, aligned using MAFFT v. 7.450 with default settings [66], and visualized by Jalview. Under-represented tetramers were detected using https://www.cmbl.uga.edu//software/signature.html (accessed on 15 December 2021), and for more complex motifs a manual search was carried out in Geneious v 8.1.9.(Auckland, New Zeeland).

The phylogenetic analyses of the adhesin and tail fiber gene sequences were carried out as follows. For both datasets, sequences were aligned using MAFFT v. 7.450 [66], and a maximum likelihood phylogenetic tree constructed with IQ-TREE v. 1.6.12 [67], using ModelFinder for model selection [68]. Additional phylogenetic analyses were conducted in BEAST 1.10.4 [69], using the best nucleotide substitution model as determined in ModelFinder and available in BEAST, namely WAG + F + G4 [70] and BLOSUM62 + F + G4 [71] for the adhesins and tail fibers gene sequences, respectively. All sequences were considered isochronous, and the evolutionary process was reconstructed under a strict molecular clock with a fixed rate of 1 and a constant population size model [72]. The infection capability of the LR2-5 strain was considered as a discrete trait analyzed using a symmetric diffusion model [73], and the number of changes between the two states (Markov jumps) was estimated in the posterior distribution of trees [74]. Proper convergence and mixing (effective sample size > 200) was verified using tracer v. 1.7 [75] and the burn-in (10% of samples) was removed. The presence of a phylogenetic signal linked to the LR2-5 infection capability was tested by comparing the distribution of Markov jumps to a “null distribution” of an estimated number of Markov jumps computed by a set of 10 independent runs, where the states (i.e., LR2-5 infection capability) were randomized. A clear overlap between the 95% highest posterior density (95% HPD) of the estimated number of Markov jumps in the original dataset and the randomized “null” distributions would sign the absence of a phylogenetic signal [73]. If not otherwise stated, default parameters were used.

## 3. Results and Discussion

### 3.1. Detection of Viruses Infecting Haloferax Strains

**Figure 1:**
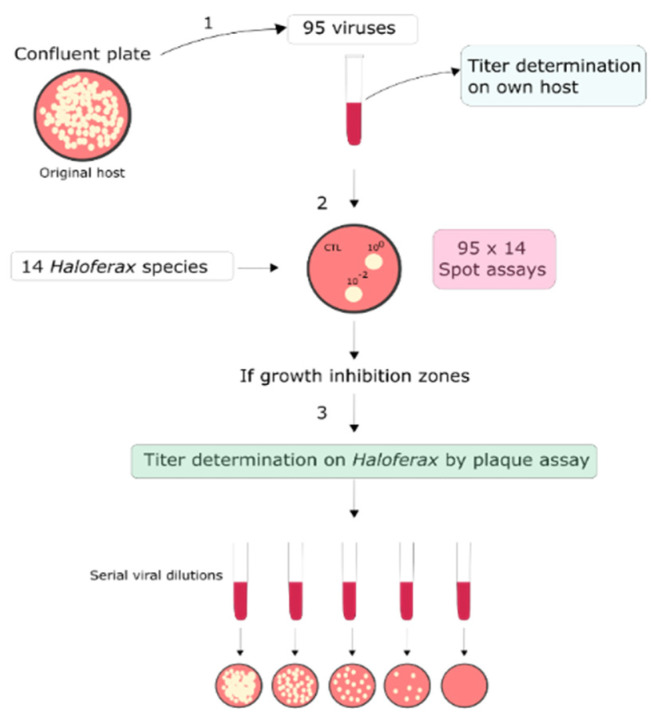
A schematic overview of the virus–host screen. **Step 1**: Fresh virus stocks made from confluent or semi-confluent plates were prepared on their own host strains and the titers were determined on their own host strains. **Step 2:** 95 virus stocks (undiluted and 10^−2^ dilution) were spotted on lawns of 14 *Haloferax* strains. MGM medium was used as a negative control (CTL). **Step 3:** After incubation at 37 °C, all virus-*Haloferax* pairs that resulted in growth inhibition on the spot-on lawn-assay were further tested by plaque assay by making serial dilutions of the virus stock and plating with the *Haloferax* strains to be tested. Viral plaques observed on *Haloferax* were counted, the titers were determined, and positive virus-*Haloferax* pairs were noted in Table 1.

To identify novel virus–host models for *Haloferax*, a collection of 95 haloarchaeal viruses (Supplementary Table S2) were cross-tested with 14 *Haloferax* strains (Supplementary Table S1, Figure 1). The viruses were isolated from samples taken from different hypersaline environments, and they are part of the collection at the University of Helsinki. Information on the origins, host strains, virus morphologies, genomes, and taxonomic classification of the viruses can be found in Supplementary Table S2. Some of the 14 tested *Haloferax* strains are widely used laboratory models, such as *Hfx. volcanii* H26 and *Hfx. mediterranei* [76], while several other strains were isolated quite recently from the hypersaline Lake Retba in Senegal (Supplementary Table S2) [59]. A scheme of the experimental pipeline for the screening is depicted in Figure 1.

The screening resulted in the detection of 10 virus isolates that could make plaques on the *Haloferax* strains tested (Table 1). Curiously, *Hfx. gibbonsii* LR2-5 was the only susceptible *Haloferax* strain. The LR2-5 infecting viruses belong to the genera *Haloferacalesvirus*, *Mincapvirus* (family *Hafunaviridae*) or *Retbasiphovirus* (family *Haloferuviridae*) (Table 1). It is important to note that during the course of the study, HCTV-6 and HCTV-13 genome sequences were reported to be identical [10], and, as a result, nine unique virus isolates infecting *Haloferax* strains were identified. Furthermore, the infectivity of the closely related virus isolates assigned to the three genera were re-tested with LR2-5 by plaque assay, but no more interactions were detected (Table 1).

In several cases, however, we observed a growth inhibition zone by spot-on-lawn assay but no plaques during the plaque assay (Table S2). As haloarchaea are known to produce antimicrobial toxins, i.e., halocins [77,78], we assume that halocins produced by the virus host cell are present in some of the virus stocks. These halocins would result in a growth inhibition in spot-on-lawn assays but not result in plaques. Only those virus–host pairs for which plaques were detected were marked as positive and true virus–host pairs. The quantitative plaque assay also allowed for the determination of the viral titer on the *Haloferax* host, which is an indication of the efficiency of infection. As a summary, we did not find any true virus–host pairs on any of the *Haloferax* strains, except on strain *Haloferax gibbonsii* LR2-5, which was isolated a few years ago from Lake Retba in Senegal at the same time as HFTV1 [59,60]. All LR2-5 infecting viruses had either *Halorubrum* or *Haloarcula* as their own host and their titers varied between 10^3^ and 10^10^ PFU/mL on LR2-5 (Table 1). On the other hand, we also calculated the efficiency of plating (EOP) of the viruses that were able to infect LR2-5 to compare them with their original isolation host. The efficiency of plating (EOP) of three mincapviruses HSTV-2, HRTV-2, and HCTV-6 originally grown either on *Halorubrum* or *Haloarcula* hosts had a slightly higher or around same EOP on LR2-5 than on their own host. However, the rest of the LR2-5 infecting viruses had three to six magnitudes lower EOPs on LR2-5 than on their own host. These results are in accordance with previous observations, which showed that myoviruses infecting halophilic archaea can have a wide host range [10,29]

### 3.2. Characteristics of Haloferax Infecting Viruses

**Figure 2:**
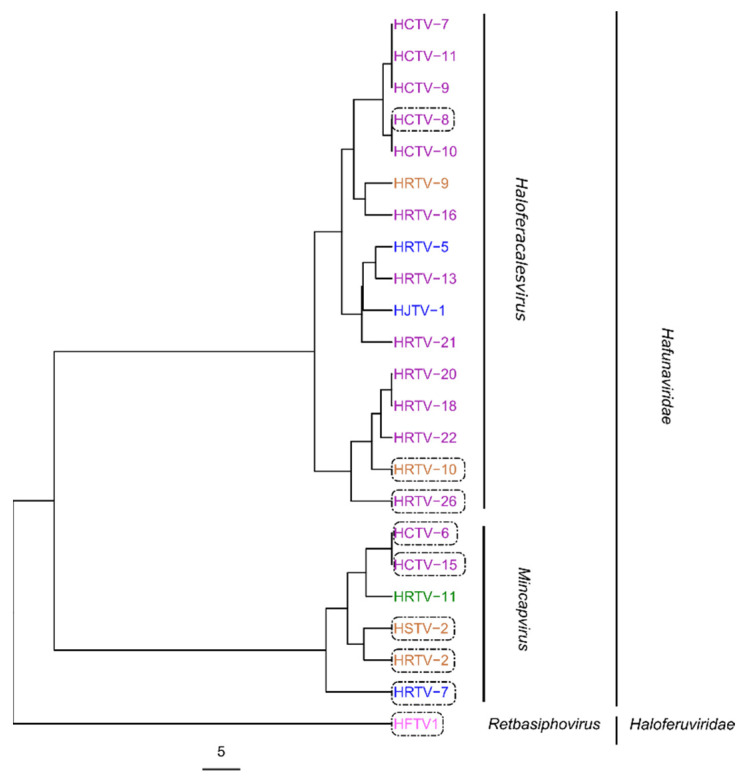
Phylogenetic tree of viruses belonging to the *Haloferuviridae* and *Hafunaviridae* families based on average nucleotide identity (ANI) values calculated using VIRIDIC software. Viruses infecting LR2-5 are surrounded by a box. Scale bar represents the number of substitutions per nucleotide position. Place of isolation: pink Senegal, purple Thailand, green Slovenia, orange Israel, and blue Italy.

To map the determinants of viral host range, we focused on viruses that infect LR2-5 and those viruses belonging to the same genus as LR2-5 infecting viruses (genera *Haloferacalesvirus*, *Mincapvirus*, *Retbasiphovirus*; Table 1). A phylogenetic tree based on the complete genome sequences of the haloferacalesviruses, mincapviruses, and retbasiphovirus (Figure 2) and their EOPs on LR2-5 (Table 1) revealed that all mincapviruses, except HRTV-11, can infect LR2-5.

**Figure 3:**
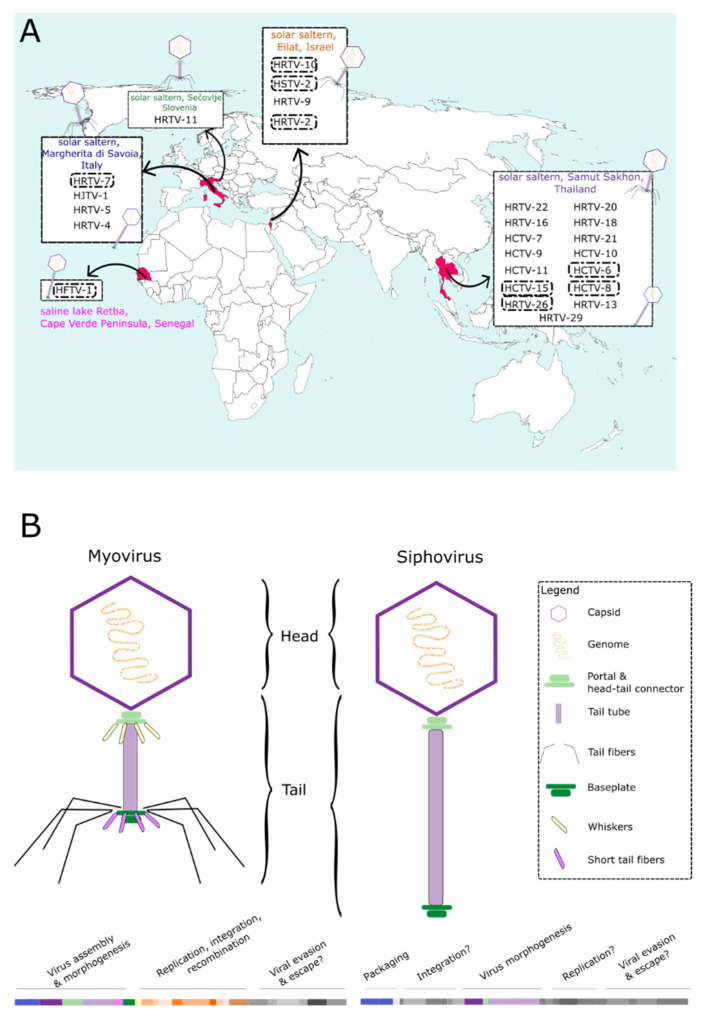
(**A**) Isolation sites of the haloferacalesviruses, mincapviruses, and retbasiphovirus (see also Supplementary Table S2). Schemes of the viral morphologies that were observed in each group are also indicated (see also Table 1). LR2-5 infecting viruses are circled with a dash line. (**B**) Schematic representation of the myovirus and siphovirus virion morphologies (not in scale) and a typical genome organization consisting of different functional modules of tailed archaeal viruses.

The LR2-5 infecting viruses have been isolated from hypersaline environmental samples either from Israel, Italy, Senegal, Slovenia, or Thailand (Figure 3A, Supplementary Table S2). All viruses represented tailed morphologies (Table 1). HFTV1 is the only siphovirus and the rest of the LR2-5 infecting viruses are myoviruses (Table 1). A schematic of the morphologies and a typical genome organization of myovirus and siphovirus are shown in Figure 3B.

HRTV-11 is the only mincapvirus isolated from a remote location, Slovenia, where no other haloarchaeal virus were successfully isolated (Figure 3A; [29]). The broad host range of myoviruses has been linked to their ability to exchange their host-specific genetic modules for receptor binding proteins [19]. Thus, we hypothesize that the host range of viruses isolated from locations with a low virus-density might be different from other closely related viruses isolated from virus-dense environments, as the possibility for recombination events might have been limited. We had a closer look into possible host range determining factors to find potential explanations for the few haloferacalesviruses that exceptionally do infect LR2-5.

### 3.3. Restriction–Modification Systems

Viruses are known to develop strategies to escape host defense mechanisms. Thus, we explored in more detail the antiviral defense mechanisms of the host and the viral escape mechanisms to determine if any of these factors could explain the differences in the ability to infect LR2-5 between viruses from the same family. LR2-5 does not have a CRISPR-cas system but encodes a predicted type I restriction modification (RM) system [60]. These antiviral mechanisms are based on methylation of host DNA (to protect it) and cleavage of unmethylated DNA (viral DNA). Part of the LR2-5 encoded RM system are a Zim methylase (CTAG methyltransferase), a Mrr-like endonuclease, and an RmeRMS (type I restriction enzyme restriction/methylation/specificity subunit).

One strategy of viruses to escape host recognition is the avoidance of certain motifs in their genomes, which are the targets for the RM systems [79]. In line with previous studies [79], the palindromic tetrameric motifs CTAG, GATC, and AGCT are absent in the mincapvirus genomes, whereas haloferacalesviruses lack only CTAG and GATC, except for HRTV-22, which contains a CTAG motif (Supplementary Table S3). Furthermore, mincapviruses and haloferacalesviruses show an under-representation of the TGCA and the CATG motifs. On the other hand, those tetrameric motifs are also under-represented in the genomes of haloferuviruses (Supplementary Table S3). We did not observe any differences between the viruses with respect to under-represented motifs, which would be able to explain the different infectivity of the studied viruses on LR2-5.

Methylated motifs in the genome of LR2-5 were previously predicted [60]. We searched for sequences in viral genomes that might be recognized by the host methyltransferases. We hypothesize that when the viral genome is replicated, the host will recognize those sequences as “self” and methylate the viral DNA, hence, allowing the virus to escape the antiviral-mechanism. Calculation of the frequency of corresponding motifs in the viral genomes revealed that the motif GCGCTG is found more frequently in all mincapviruses than in haloferacalesviruses (Supplementary Table S4). The frequency of the other motifs was similar for all viruses. Therefore, we concluded that host range determinants in case of LR2-5 infecting viruses might rely on another factor.

### 3.4. Adhesins and Tail Fiber Proteins

**Figure 4:**
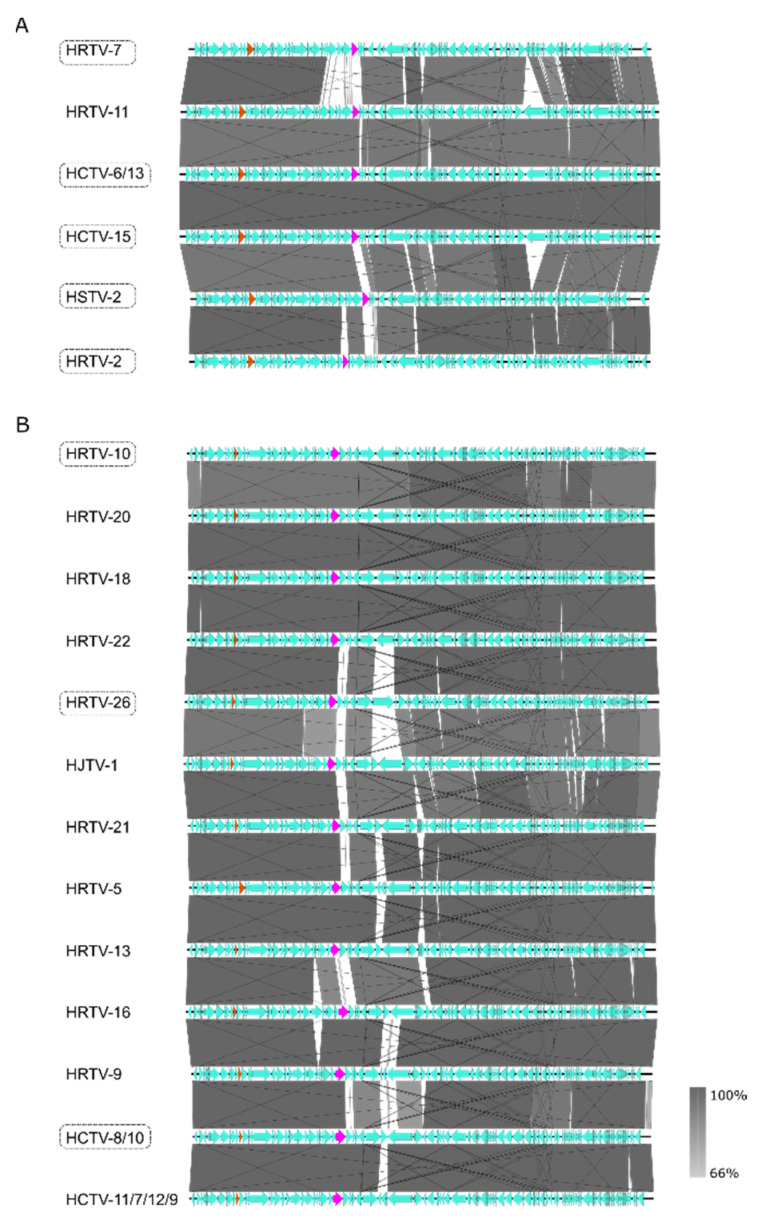
Schematic genomic alignment of the (**A**) mincapviruses, and (**B**) haloferacalesviruses. Grey bars represent homologous genomic regions. The level of nucleotide identity is reflected by the intensity of grey. Genes encoding major capsid protein (orange) and adhesins (pink) are indicated. The LR2-5 infecting viruses are circled with dashed lines. Identical or very similar genomes are shown as one and virus names are separated by /. Figures are prepared with Easyfig.

Pairwise alignment of the viral genomes of the *Hafunaviridae* family members showed the same genome organization within their own group (mincapviruses or haloferacalesviruses) (Figure 4). There were only a few variable genomic regions observed. Firstly, at the right-end of the genomes, a region was detected with frequent insertions and inversions (Figure 4). Many of the proteins encoded by genes in this region are designated as hypothetical and they lack homology with proteins found in reference databases. It might be possible that these genes encode proteins involved in viral egress, as several of them have predicted transmembrane domains. However, since egress mechanisms of haloarchaeal viruses are not well understood, and the responsible proteins are not identified, it is not clear if these genes are determinants of differences observed in viral infectivity on LR2-5.

**Figure 5:**
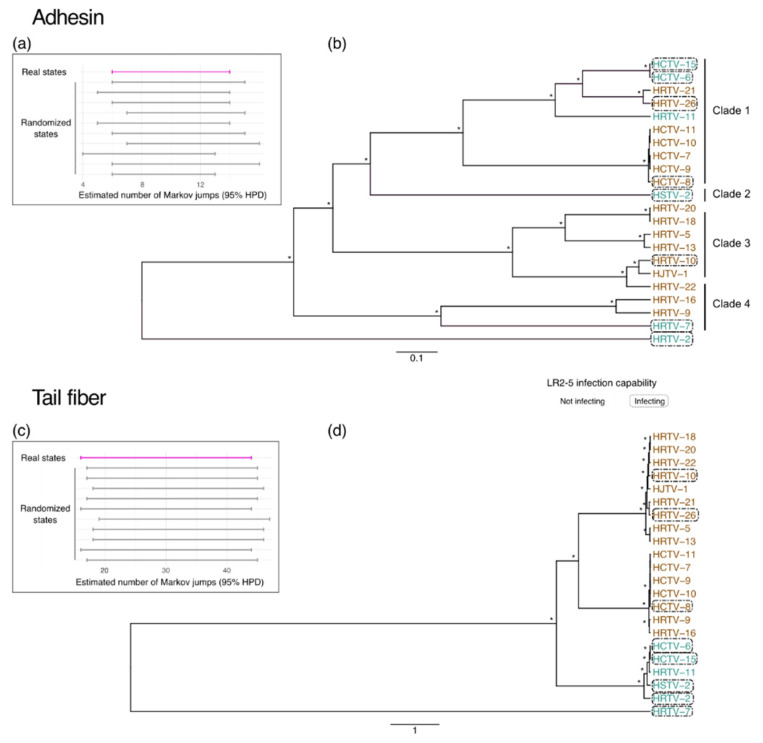
Comparison of the adhesin and tail fiber gene sequence phylogenies to the ability of viruses to infect LR2-5. (**a**,**c**) Distribution of Markov jumps (95% HPD) for (i) real states (pink) and (ii) randomized states (grey). (**b**,**d**) Bayesian maximum clade credibility tree with discrete trait reconstruction based on adhesin or tail fiber gene sequences, respectively. Asterisk indicates node posterior probabilities higher than 0.8, and viruses in the boxes can infect LR2-5. Color code indicates the genus; blue *Mincapvirus*, brown *Haloferacalesvirus*.

Another highly variable region in the viral genomes is located around genes encoding the viral tail fiber and adhesin (Figure 4 and Figure 5). Adhesins, located at the distal tip of the tail fibers, have been shown to be the host determinants in the tailed dsDNA bacteriophages of the order *Caudoviricetes* [17]. Long tail fibers of bacteriophages have a modular organization, and they consist of several different proteins [18,80]. Moreover, it has been suggested that haloarchaeal tailed viruses exchange the genes encoding either the tail fiber or adhesin with other viruses by recombination [10]. All haloferacalesviruses and mincapviruses have a gene encoding for a putative tail fiber protein together with a gene encoding a putative adhesin [10]. HFTV1 is a siphovirus with a long non-contractile tail, and it does not contain tail fibers. 

A tree of the hafunaviruses (haloferacalesviruses and mincapviruses) tail fiber adhesin proteins showed that they are divided in four clades (Figure 5), as also shown by Liu et al. [10]. Clade 1 and Clade 3 adhesins were previously reported to correlate well with the observed virus host range among the tested *Haloarcula*, *Halobacterium*, *Halobellus*, *Halorubrum*, and *Haloterrigena* strains [10]. We aimed to test if this correlation between adhesins and host range could also be seen in our current data set. 

We investigated the hypothetical relationship between the susceptibility of the LR2-5 strain to infection and diversification of the hafunavirus tail fiber and adhesin genes by comparing the number of Markov jumps (i.e., infecting or not LR2-5) through the adhesin and the tail fiber gene evolution (Figure 5). A “null” distribution for each gene was generated by randomizing the state’s distribution. We observed a clear overlap between the estimated numbers of Markov jumps (95% highest posterior density; HPD) in the real and the randomized datasets, indicating the lack of a phylogenetic signal linked to the viral infection capability on the LR2-5 strain for both the hafunavirus tail fiber gene and the adhesin gene (Figure 5).

Based on this analysis, we did not observe any clear correlation between the type of adhesin or tail fiber gene and the viral ability to infect *Hfx. gibbonsii* LR2-5. However, it should be emphasized that our dataset was limited to *Haloferax* hosts. Such a correlation between host range and adhesin type might only become apparent when a more diverse set of hosts is used, as the *Haloferax* strains are generally not very susceptible for viral infection. However, we did find a few case examples, which seems to pinpoint the adhesin as a host determining factor. First, HRTV-26, one of the few haloferacalesviruses that infects LR2-5, encodes a Clade 1 adhesin, similar to most mincapviruses. Since the adhesins of haloferacalesviruses that are closely related to HRTV-26 all belong to Clade 3, it is likely that HRTV-26 picked up an adhesin gene via horizontal gene transfer from one of the Clade 1 viruses. This might be the explanation for why HRTV-26, even though it is an haloferacalesvirus, is still capable of infecting LR2-5.

Second, viruses encoding a Clade 3 adhesin do not infect LR2-5, with the exception of HRTV-10. To gain more insight into the HRTV-10 tail fiber adhesin, a multiple sequence alignment was performed. We identified one mutated codon resulting in an amino acid change (T380A) in a conserved motif at the 3′end of the adhesin gene (Supplementary Figure S2). This subtle substitution might be a possible explanation for its increased infectivity on LR2-5, as this could produce alterations in the fold and the topology of adhesins. Liu et al. [10] already discussed how a single amino acid substitution (A217V) in the adhesins of HRTV-19 and HRTV-23 alters the host range. Moreover, Trojet et al. [17] showed how the long tail fiber locus in the T4 superfamily viruses is susceptible to frequent modular shuffling, which results in chimeric adhesins and, thus, viruses acquire new host receptor specificities. However, this hypothesis requires further experiments.

## 4. Conclusions

We aimed to develop an euryarchaeal virus–host model system with attractive molecular, genetic, and imaging tools to dive deeper into virus–host relationships in archaea. We focused on *Haloferax* strains, as the most advanced molecular and genetic tools are available for these organisms. Due to the low number of known viruses that infect *Haloferax*, we set up a large-scale assay to identify viruses capable of infecting *Haloferax* strains. Our extensive screening of approximately 100 isolated and mostly characterized haloarchaeal viruses showed that only a small subset was capable of infecting *Haloferax*. Specifically, *Hfx. gibbonsii* LR2-5 showed itself as an auspicious host, as we identified 10 virus isolates that can infect this host and all other hosts could not be infected. Previously, we studied potential factors that make *Hfx. gibbonsii* LR2-5 (DSM No. 112399) so much more receptive to virus infection in contrast to related strains. For its virus susceptibility, several possible factors were identified as potential explanations. The most prominent was an absence of CRISPR-cas virus defense systems in LR2-5 [60]. In addition, *Hfx. gibbonsii* LR2-5 has a different RM system from some other *Haloferax* strains, and the cell surface might show a different composition than that of other *Haloferax* strains because LR2-5 encodes divergent surface proteins that might serve as viral receptors, such as pili and S-layer proteins [60]. These latter two proteins are a common component of the archaeal cell-surface, and they are hypothesized to be used by viruses for initial binding and recognition [81,82].

Because we used plaque assays for the host range determination, we cannot distinguish at which step of the infection cycle viruses are not successful in infecting a host: entry, replication, or release. However, the results of the present study might indicate that adhesins, which are required for entry, are an important factor determining host range specificity, whereas other factors such as the viral egress proteins and restriction–modification systems might also play a role. Moreover, it should be noted that one limitation of the approach utilized is the assumption that the successful infection of the host results in plaque formation. It could be that some of the viruses studied here might have integrated their genome when infecting *Hfx. gibbonsii* LR2-5 and transferred to a lysogenic cycle. Consequently, these types of interactions would not show clear plaques, and they could have been missed by our approach. However, further experimental research on the host range implications of these proteins is needed.

Our analysis has identified *Hfx. gibbonsii* LR2-5 as the only virus susceptible *Haloferax* strain. This host can be infected by 10 different viruses with sequenced genomes [10,16], and it thus offers the possibility to compare infection mechanisms between viruses. Moreover, its genome is sequenced and its cell biology is characterized [60]. We are also currently developing a genetic system based on PyrE for *Hfx. gibbonsii* LR2-5. We conclude that this species is an extremely promising model host to study virus–host interactions in haloarchaea, and we anticipate that this work serves as a stepping stone for future in-depth molecular characterization of archaeal viral infection mechanisms.

## Figures and Tables

**Table 1:** *Haloferax gibbonsii* LR2-5 infecting viruses and the closely related virus isolates assigned to the same genus.

Virus	Virus Morphology ^(5)^	Host Strain to Grow the Virus	Titer on Own Host, pfu/mL	Titer on LR2-5, pfu/mL	EOP on LR2-5 ^(6)^
*Hafunaviridae* (F) ^(1)^					
*Haloferacalesvirus* (G) ^(2)^					
HRTV-10	M	*Halorubrum* sp. B2-2	1.2 × 10^9^	6.8 × 10^3^	6 × 10^−6^
HRTV-18	M	*Halorubrum* sp. SS10-3	7.5 × 10^9^	-	
HRTV-20	M	*Halorubrum* sp. SS10-9	7.0 × 10^10^	-	
HRTV-22	M	*Halorubrum* sp. SS10-9	1.1 × 10^10^	-	
HRTV-26	M	*Halorubrum* sp. SS10-9	1.4 × 10^9^	4.3 × 10^6^	3 × 10^−3^
HRTV-5	M	*Halorubrum* sp. s5a-3	2.0 × 10^10^	-	
HCTV-7 ^(3)^	M	*Haloarcula californiae*	4.8 × 10^10^	-	
[HCTV-12] ^(3)^	M	*Haloarcula californiae*	1.3 × 10^10^	-	
HCTV-9	M	*Haloarcula californiae*	2.8 × 10^10^	-	
HCTV-11	M	*Haloarcula californiae*	4.0 × 10^10^	-	
HRTV-9	M	*Halorubrum* sp. B2-2	5.3 × 10^9^	-	
HRTV-16	M	*Haloterrigena* sp. SS13-7	nd	nd	
HCTV-8	M	*Haloarcula californiae*	2.3 × 10^10^	3.1 × 10^5^	1 × 10^−5^
HCTV-10	M	*Halorubrum sodomense*	2.3 × 10^9^	-	
HJTV-1	M	*Haloarcula japonica*	1.6 × 10^9^	-	
HRTV-13	M	*Halorubrum* sp. SS8-2	5.1 × 10^9^	-	
HRTV-21	M	*Halorubrum* sp. SS10-9	2.8 × 10^9^	-	
*Mincapvirus* (G) ^(2)^					
HSTV-2	M	*Halorubrum sodomense*	8.2 × 10^9^	2.3 × 10^10^	3
HRTV-7	M	*Halorubrum* sp. B2-2	2.1 × 10^9^	7.9 × 10^5^	4 × 10^−4^
HRTV-2	M	*Halorubrum* sp. s1-2	2.0 × 10^10^	4.6 × 10^10^	2
HRTV-11	M	*Halorubrum* sp. SL-5	4.7 × 10^10^	-	
HCTV-6 ^(4)^	M	*Haloarcula californiae*	1.6 × 10^10^	3.9 × 10^9^	2 × 10^−1^
[HCTV-13] ^(4)^	M	*Haloarcula californiae*	2.5 × 10^9^	[1.3 × 10^7^]	[5 × 10^−3^]
HCTV-15	M	*Halorubrum* sp. SS6-2	1.9 × 10^10^	9.6 × 10^6^	5 × 10^−4^
*Haloferuviridae* (F) ^(1)^					
*Retbasiphovirus* (G) ^(2)^					
HFTV1	S	*Haloferax gibbonsii* LR2-5	2.9 × 10^12^	2.9 × 10^12^	1

^(1)^ Family; ^(2)^ Genus; ^(3)^ HCTV-7 and HCTV-12 are identical and both were tested here; HCTV-7 will be used in future; ^(4)^ HCTV-6 and HCTV-13 are identical and both were tested here; HCTV-6 will be used in future; ^(5)^ S, siphovirus morphology; M, myovirus morphology; ^(6)^ Relative efficiency of plating (EOP) on LR2-5 compared to the own host with an EOP of 1.

## Data Availability

The data presented in this study are available within the article and its Supplementary Materials.

## Supplementary Materials

The following supporting information can be downloaded at: https://www.mdpi.com/article/10.3390/v14061344/s1, Table S1. *Haloferax* strains used in the study [19,29,37,59,60,76]; Table S2. Viruses used in the study; Table S3. Underrepresented palindromic motifs in viral genomes; Table S4. Manual counting of modified DNA motifs found in the genomes of viruses infecting *Hfx. gibbonsii* LR2-5; Figure S1. Multiple sequence alignment of adhesins belonging to the Group 3, Figure S2. VIRIDIC generated heatmap.
