# Supplementary material for: The Viral Susceptibility of the Haloferax Species
Source: Viruses. 2022 Jun 20;14(6):1344. doi: 10.3390/v14061344 (PMC9229481; doi:10.3390/v14061344)
Supplement: Supplementary file 1 [file viruses-14-01344-s001.zip › viruses-1750812-supplementary (1)/220615 Supplementary_ZA.pdf]

Supplementary material

Viral susceptibility of *Haloferax* species

Zaloa Aguirre Sourrouille [1*], Sabine Schwarzer [1,2*], Sebastian Lequime [3], Hanna M. Oksanen [4#] and Tessa E.F. Quax [1,2#]

[1] Biology of Archaea and Viruses, Groningen Biomolecular Sciences and Biotechnology Institute, University of Groningen, 9747 AG Groningen, The Netherlands.

[2] Archaeal Virus-Host Interactions, Faculty of Biology, University of Freiburg, Freiburg, Germany

[3] Cluster of Microbial Ecology, Groningen Institute for Evolutionary Life Sciences, University of Groningen, Groningen, the Netherlands

[4] Molecular and Integrative Biosciences Research Programme, Faculty of Biological and Environmental Sciences, University of Helsinki, Helsinki, Finland.

Supplementary Table S1. *Haloferax* strains used in the study

Supplementary Table S2. Viruses used in the study

Supplementary Table S3. Underrepresented palindromic motifs in viral genomes

Supplementary Table S4. Manual counting of modified DNA motifs found in the genomes of viruses infecting *Hfx. gibbonsii* LR2-5

Supplementary Figure S1. Multiple sequence alignment of adhesins belonging to the Group 3

Supplementary Figure S2. VIRIDIC generated heatmap

Supplementary References

Table S1. ***Haloferax*** **strains used in the study**

Strain	Reference
Haloferax volcanii H26	[37]
Haloferax sp. s5a-1	[29]
Haloferax sp. SP10-1	[29]
Haloferax sp. SS9-6	[19]
Haloferax sp. SS10-6	[19]
Haloferax sp. SS10-7	[19]
Haloferax sp. LR1-5	[59]
Haloferax sp. LR1-14	[59]
Haloferax sp. LR1-18	[59]
Haloferax sp. LR1-19	[59]
Haloferax sp. LR1-24	[59]
Haloferax gibbonsii LR2-5	[59,60]
Haloferax sp. LR2-16	[59]
Haloferax mediterranei DSM 1411	[76]

Table S2. Viruses used in the study

Please see separate excel table

Table S3. Underrepresented palindromic motifs in viral genomes[1]

	CTAG	GATC	AGCT	TGCA	CATG
HFTV1	0.0314	0.0714	0.2252	0.8473	0.9074
HCTV-6	0	0	0	0.2976	0.4192
HCTV-15	0	0	0	0.2976	0.4192
HRTV-11	0	0	0	0.2742	0.3604
HSTV-2	0	0	0	0.3023	0.3638
HRTV-7	0	0	0	0.3291	0.3654
HRTV-2	0	0	0	0.3091	0.3721
HRTV-10	0	0	n	0.2756	0.3855
HRTV-20	0	0	n	0.2959	0.3261
HRTV-18	0	0	n	0.2964	0.3263
HRTV-26	0	0	n	0.2764	0.3341
HRTV-22	0.0067	0	n	0.2707	0.3146
HJTV-1	0	0	n	0.24	0.3905
HRTV-21	0	0	n	0.2565	0.3741
HRTV-5	0	0	n	0.2757	0.3566
HRTV-13	0	0	n	0.2604	0.3638
HRTV-16	0	0	n	0.2811	0.3554
HRTV-9	0	0	n	0.3411	0.3623
HCTV-10	0	0	n	0.2886	0.3382
HCTV-8	0	0	n	0.2886	0.3379
HCTV-7	0	0	n	0.2993	0.3408
HCTV-11	0	0	n	0.2994	0.3412
HCTV-9	0	0	n	0.2991	0.3409

[1] Calculated as Odds Markov values where they are not zero. n represent normal frequencies. Orange color incidates the value 0.

Table S4 Manual counting of modified DNA motifs found the genomes of viruses infecting *Hfx. gibbonsii* LR2-5

	**C**TAG [1]	GCG**C**TG	**A**YCnnnnnCTTYG	CRA**A**GnnnnnGRT	CC**A**nnnnnnRTCnC	GnG**A**YnnnnnnTGG
HFTV-1	2	0	2	0	3	9
HCTV-6	0	41	9	14	6	10
HCTV-15	0	41	9	14	6	10
HRTV-11	0	39	6	15	9	7
HSTV-2	0	37	8	14	8	6
HRTV-7	0	32	6	15	9	5
HRTV-2	0	36	8	15	8	9
HRTV-10	0	12	8	10	5	6
HRTV-20	0	14	11	11	7	6
HRTV-18	0	14	11	11	7	6
HRTV-26	0	16	12	9	7	4
HRTV-22	1	13	13	9	6	5
HJTV-1	0	9	9	10	4	9
HRTV-21	0	13	9	10	3	7
HRTV-5	0	10	5	11	5	7
HRTV-13	0	10	6	11	5	8
HRTV-16	0	10	6	10	2	7
HRTV-9	0	14	5	12	3	9
HCTV-10	0	10	5	10	5	7
HCTV-8	0	10	5	10	5	7
HCTV-7	0	10	6	10	5	7
HCTV-11	0	10	6	10	5	7
HCTV-9	0	10	6	10	5	7

[1] Modified bases are shown in bold. The color range blue to yellow is from a low number of occurrences (0) to a high number of occurrences (>30)

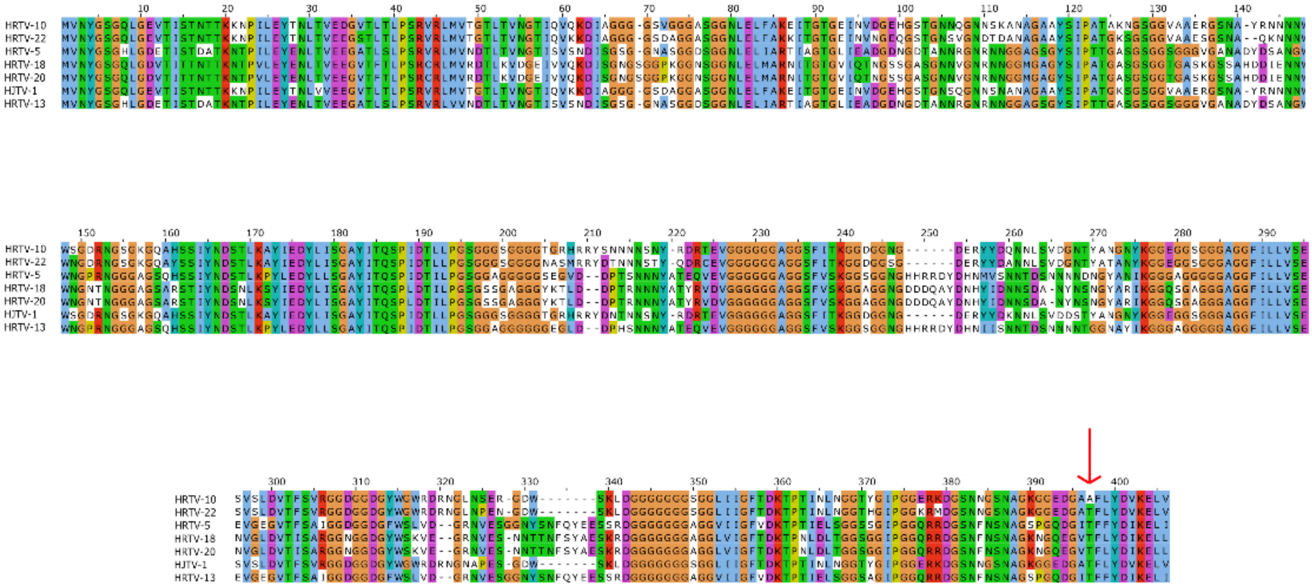

Figure S1. Multiple sequence alignment of adhesins belonging to the Clade 3 (see also Figure 5). Protein sequences were aligned using Clustal W. The colors correspond to the amino acid identity. The arrow indicates the T380A substitution in HRTV-10.

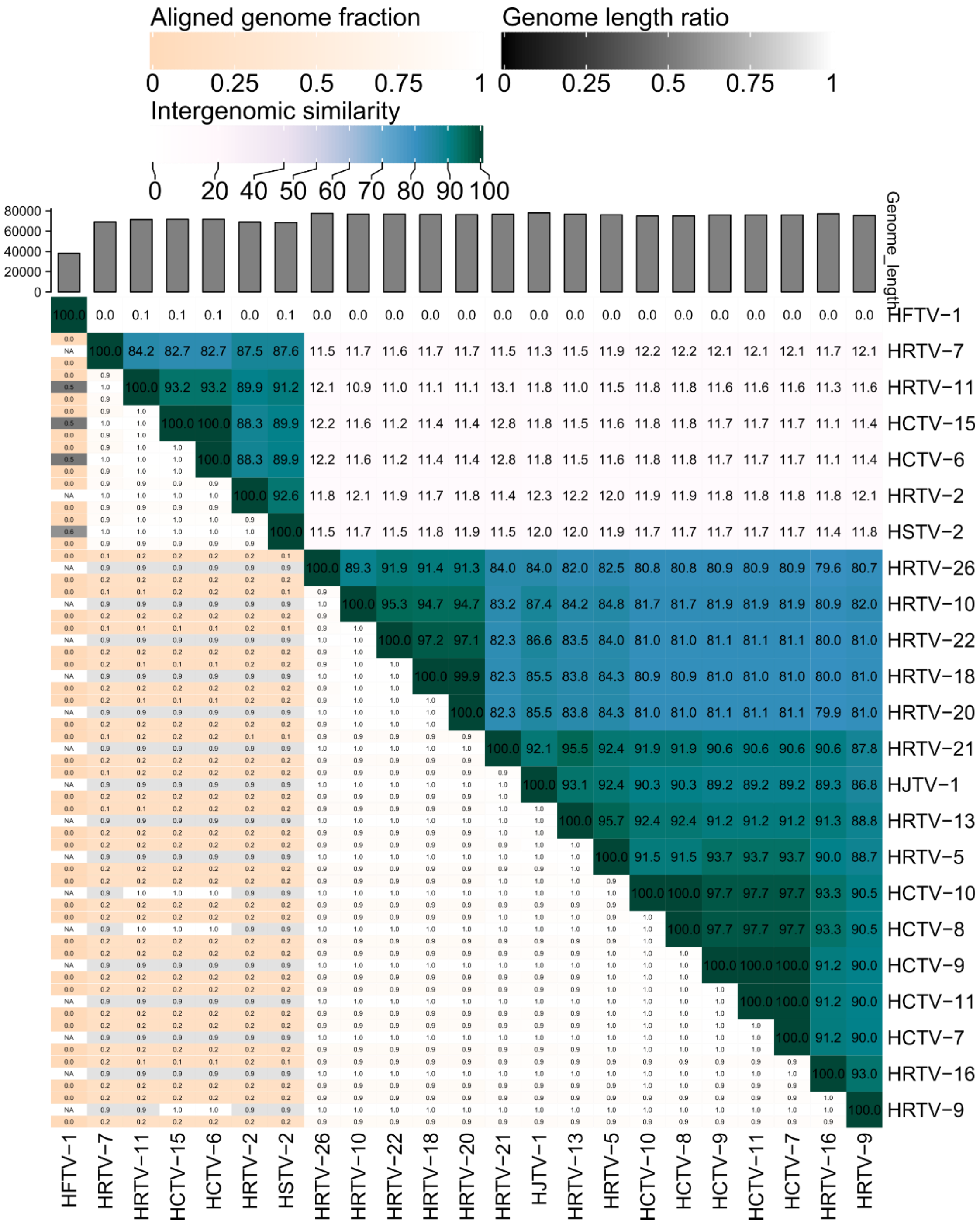

Figure S2. VIRIDIC generated heatmap. The right half indicates the intergenomic similarity between the viral genomes using a color gradient. Darker colors indicate higher intergenomic similarities. The left side shows three different values; the aligned genome fraction for the genome found in the row(top), genome length ratio for the genomes in this pair (middle) and the aligned genome fraction for the genome found in this column (bottom).
